# Predictors of improvement in quality of life at 12-month follow-up in patients undergoing anterior endoscopic skull base surgery

**DOI:** 10.1371/journal.pone.0272147

**Published:** 2022-07-27

**Authors:** Quinlan D. Buchlak, Nazanin Esmaili, Christine Bennett, Yi Yuen Wang, James King, Tony Goldschlager

**Affiliations:** 1 School of Medicine, The University of Notre Dame Australia, Sydney, NSW, Australia; 2 Department of Neurosurgery, Monash Health, Melbourne, VIC, Australia; 3 Faculty of Engineering and Information Technology, University of Technology Sydney, Ultimo, NSW, Australia; 4 St Vincent’s Hospital, Melbourne, VIC, Australia; 5 Royal Melbourne Hospital, Melbourne, VIC, Australia; 6 Department of Surgery, Monash University, Melbourne, VIC, Australia; PLOS: Public Library of Science, UNITED KINGDOM

## Abstract

**Background:**

Patients with pituitary lesions experience decrements in quality of life (QoL) and treatment aims to arrest or improve QoL decline.

**Objective:**

To detect associations with QoL in trans-nasal endoscopic skull base surgery patients and train supervised learning classifiers to predict QoL improvement at 12 months.

**Methods:**

A supervised learning analysis of a prospective multi-institutional dataset (451 patients) was conducted. QoL was measured using the anterior skull base surgery questionnaire (ASBS). Factors associated with QoL at baseline and at 12-month follow-up were identified using multivariate logistic regression. Multiple supervised learning models were trained to predict postoperative QoL improvement with five-fold cross-validation.

**Results:**

ASBS at 12-month follow-up was significantly higher (132.19,SD = 24.87) than preoperative ASBS (121.87,SD = 25.72,p<0.05). High preoperative scores were significantly associated with institution, diabetes and lesions at the planum sphenoidale / tuberculum sella site. Patients with diabetes were five times less likely to report high preoperative QoL. Low preoperative QoL was significantly associated with female gender, a vision-related presentation, diabetes, secreting adenoma and the cavernous sinus site. Top quartile change in postoperative QoL at 12-month follow-up was negatively associated with baseline hypercholesterolemia, acromegaly and intraoperative CSF leak. Positive associations were detected for lesions at the sphenoid sinus site and deficient preoperative endocrine function. AdaBoost, logistic regression and neural network classifiers yielded the strongest predictive performance.

**Conclusion:**

It was possible to predict postoperative positive change in QoL at 12-month follow-up using perioperative data. Further development and implementation of these models may facilitate improvements in informed consent, treatment decision-making and patient QoL.

## Introduction

Pituitary adenomas are common and may be present in up to 10% of people with normal endocrine function [1]. Prevalence ranges between 1 in 865 and 1 in 2,688 adults [2]. Functioning pituitary tumours lead to hypersecretion syndromes including Cushing’s disease, acromegaly and hyperprolactinemia [3], negatively impacting patient function, life expectancy and quality of life (QoL) [4]. Non-functioning tumours may lead to symptoms due to mass effect and may present with visual disturbance [1]. Treatment is provided by experienced, specialised multidisciplinary centres, which include neurosurgery, endocrinology, rhinology, radiology and radiation oncology [2].

Patients with pituitary lesions experience worse QoL than the general population [3, 5, 6]. Treatment aims to improve QoL or at least arrest its decline, although postoperative QoL typically declines initially post-surgery [7] before improving. However, persistent decline has been reported [6, 8]. Subtotal resection has been linked with QoL decrements, suggesting the need for gross total resection [9]. Endoscopic pituitary surgery has been linked with preserved patient QoL, compared to other surgical techniques [9, 10]. If future postoperative QoL improvement could be accurately predicted prior to pituitary surgery, then this could provide valuable decision support information to clinicians and patients, which may lead to altered, more personalised treatment plans and clearer postoperative recovery expectations. Clinicians could determine which patients would be most likely to experience QoL improvements and patients with poor predicted QoL outcomes could be considered for alternative or adjunct treatments.

Supervised machine learning is a subdomain of artificial intelligence (AI) that involves the application of algorithms to identify complex patterns in large datasets enabling effective outcome prediction and classification [11–17]. Supervised learning techniques have demonstrated efficacy across a wide range of application domains and the application of machine learning to neurosurgery is growing rapidly [14, 18, 19]. With regard to skull base surgery, supervised learning has been applied to predict early postoperative outcomes [20], hyponatremia [21], the risk of experiencing intraoperative cerebrospinal fluid (CSF) leaks [22], remission after surgery [23, 24] and long-term postoperative control of Cushing’s disease [25]. It has been used to classify adenoma subtypes using magnetic resonance imaging data [26] and predict radiotherapeutic response in patients with acromegaly [27].

This study was conducted to detect clinical associations with QoL in trans-nasal endoscopic skull base surgery patients and train and test a collection of supervised learning classifiers to predict QoL improvement at 12 months. The study was guided by the following two research questions. (1) What clinical factors are associated with preoperative QoL in skull base surgery patients? (2) Can postoperative change in QoL be effectively predicted using supervised learning algorithms?

## Method

### Design

This multi-institutional study involved an analysis of a prospectively collected dataset. Each patient was older than 18 years and underwent skull base neurosurgery to treat pituitary pathology. Patients were treated at three tertiary hospitals in Melbourne, Australia: St Vincent’s Hospital, Monash Medical Centre, and The Royal Melbourne Hospital.

### Ethics

The study was conducted under institutional review board (IRB) ethics approval (2021-029S, The University of Notre Dame Australia). Patients provided their consent for the use of their data for quality improvement analysis. The IRB provided a waiver of consent for the use of the deidentified dataset for research.

### Data

#### Covariates and outcomes

Covariates (i.e., independent variables or features) included operating institution, gender, age, presentation history, co-morbidities, anatomic site of the lesion, histopathology, endocrine status, characteristics of the surgery, and intra- and postoperative complications. The primary outcome measure was QoL, as measured by the Anterior Skull Base Surgery Questionnaire (ASBS). Scores range from 35 to 175 and higher scores indicate a better state of QoL [28, 29]. Preoperative ASBS scores for each patient were stratified into quartiles. Statistical models were designed to detect associations with the highest and lowest preoperative ASBS quartiles. Postoperative change in ASBS score at 12-months was calculated for each patient and stratified into quartiles. Statistical models were designed to detect clinical associations with the highest and lowest ASBS change quartiles and machine learning models were trained and tested to predict the highest ASBS change quartile. Data were collected between March 2016 and September 2020.

#### Data processing

Covariates and outcomes were coded as binary variables. If datapoints were missing for >10% of cases for a given covariate, then it was excluded from the analysis. If <10% of cases for a binary covariate contained missing data, then it was assumed that the patient’s clinical state with regard to that covariate was normal and the missing fields were filled with zeros. This was done to retain and include as many clinical covariates as possible in the analysis, maximising the use of the specialist dataset collected. In the postoperative machine learning dataset, this applied to five covariates: “any postoperative complication” (n missing = 15), “secreting adenoma” (n missing = 8), “development of postoperative diabetes insipidus” (n missing = 10), “development of postoperative syndrome of inappropriate anti-diuretic hormone secretion (SIADH)” (n missing = 15) and “reoperation” (n missing = 4). Patients with missing outcome data were excluded from the analysis.

### Analysis

The analysis involved two phases: (1) statistical analysis using multivariate logistic regression; and (2) training and testing supervised learning classifiers. The first phase involved applying multivariate logistic regression to detect significant associations between covariates and outcome variables. Covariates were included in multivariate models in a hypothesis-driven manner based on clinical relevance and the expertise of senior surgeons [30]. Covariates with negligible statistical contribution to these multivariate models (z-score<0.02, or p>0.9) were excluded and models were subsequently retrained [15, 16, 31]. Discrete groups of related variables (e.g., demographics, presentation history factors, co-morbidities, etc.) were added sequentially and systematically to carefully assess the stability of associations. Odds ratios (OR) and 95% confidence intervals (CI) were calculated to assess the strength of associations. Predictive modelling better practice guidelines informed model development [32–35]. Highly correlated variables were removed to control for multicollinearity (e.g., “presentation history: visual” and “abnormal preoperative vision”). Logistic regression models were considered significant if they achieved a log likelihood ratio (LLR) p-value of less than 0.05.

**Fig 1:**
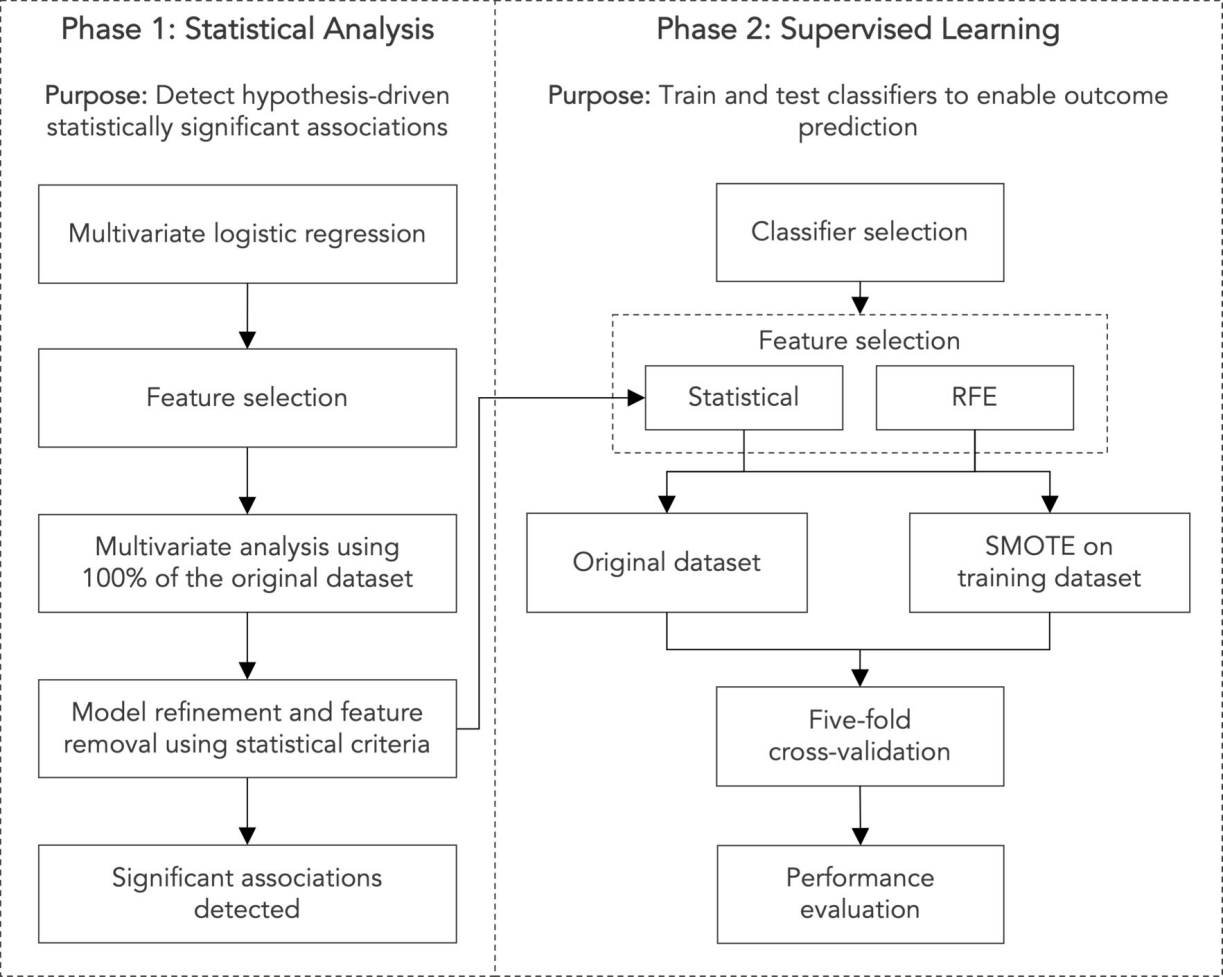
Methodology overview. RFE, recursive feature elimination. SMOTE, synthetic minority oversampling technique.

Numerous supervised learning classifiers were trained and tested, including random forest (RF) [36], gradient boosting machines (GBM) [37, 38], AdaBoost classifiers [39], support vector machines (SVM) [40], K-nearest neighbor (KNN) classifiers, gaussian naive Bayes (GNB) [41] classifiers and neural networks (NN) [12, 42–44]. Hyperparameter tuning was conducted with five-fold cross-validation. Neural networks comprised two hidden layers, the first containing 20 nodes and the second containing 10. Early stopping was implemented to mitigate overfitting. Dimensionality reduction was achieved using two methods: (1) the statistical multivariate logistic regression approach described above; and (2) recursive feature elimination (RFE) with support vector regression. The top 27 covariates were selected for inclusion. The synthetic minority oversampling technique (SMOTE) was applied to counter class imbalance in the supervised learning analysis [45]. SMOTE has been designed to avoid overfitting [46] and, as recommended, was applied to the training dataset only [47]. Discrimination between outcome classes was assessed primarily using the area under (AUC) the receiver operating characteristics (ROC) curve and the Matthews correlation coefficient (MCC). MCC is a useful metric for evaluating binary classifiers and has been presented as the preferred metric [48]. It ranges from -1 to 1, with higher scores indicating a more effective classifier. Other performance metrics included accuracy, sensitivity, specificity, positive predictive value (PPV) and F1 [49, 50]. Five-fold cross-validation was applied. Two-tailed t-tests were used to assess differences between groups. Shapley additive explanations (SHAP) were used to assess and visualise feature importance within GBM models [51]. Analyses were conducted using custom Python scripts and the statsmodels [52], SciPy [53], Scikit-learn [54], imbalanced-learn [55], Matplotlib [56], numpy [57], pandas [58] and SHAP [59] packages. Fig 1 presents a methodological overview.

## Results

A preoperative ASBS score was recorded for 451 patients. Mean patient age was 53.63 years (SD = 16.86). One hundred and ninety-nine patients had an ASBS score recorded at 12-month follow-up. Mean patient age was 52.85 years (SD = 17.31). There was no statistically significant demographic difference between the pre- and postoperative groups. Mean preoperative ASBS was 121.87 (SD = 25.72), while mean ASBS at 12 months was 132.19 (SD = 24.87), which was significantly higher (t = 4.68, p<0.05). Mean change in ASBS score at 12-month follow-up was 7.5 points (SD = 25.79). Descriptive statistics are displayed in Table 1. Most patients (n = 121) experienced a positive postoperative change in ASBS at 12-months (mean = 23.29, SD = 16.83), seventy-five patients experienced a negative change in ASBS at 12-months (mean = -17.67, SD = 16.38) and three patients experienced no ASBS change.

**Table 1:** Descriptive statistics. Covariates comprising <1% of the dataset were excluded.

Covariates	Patients with preoperative ASBS scores (n = 451)	Patients with ASBS scores at 12-month follow-up (n = 199)
Institution 1	38%	48%
Institution 2	35%	35%
Institution 3	27%	18%
Female	54%	50%
Presentation history:		
Headaches	27%	27%
Hormonal	29%	31%
Visual	40%	41%
Incidental	13%	16%
Surveillance	10%	8%
Rhinorrhea	3%	4%
Other	8%	5%
Co-morbidities:		
Atrial fibrillation	2%	3%
Asthma	3%	3%
Cancer (non-CNS)	3%	2%
Depression	4%	3%
Diabetes (non-insulin requiring)	8%	8%
Diabetes (insulin-requiring)	8%	6%
Hypertension	28%	25%
High Cholesterol	12%	12%
Osteoarthritis	3%	3%
Ulcer disease	2%	1%
Secreting adenoma	22%	22%
Preoperative endocrine function normal	54%	54%
Preoperative endocrine function deficient	18%	19%
Preoperative endocrine function hypersecreting	24%	24%
Operative factors:		
Reoperation	16%	17%
Anatomic site: sella	79%	81%
Anatomic site: suprasellar cistern	56%	59%
Anatomic site: clivus	8%	6%
Anatomic site: planum sphenoidale / tuberculum sella	8%	6%
Anatomic site: anterior fossa / olfactory groove	7%	6%
Anatomic site: sphenoid sinus	11%	9%
Anatomic site: ethmoid sinus	6%	4%
Anatomic site: cavernous sinus	7%	9%
Histopathology:		
Acromegaly	9%	9%
Gonadotroph secretory pituitary adenoma	4%	7%
Meningioma	8%	7%
Non-functioning pituitary adenoma	23%	25%
Pituitary cyst-Rathke’s cleft cyst	6%	4%
Postoperative development of diabetes insipidus	11%	12%
Postoperative development of SIADH	6%	6%
Resection: subtotal / debulk	8%	17%
Kelly intraoperative leak: Moderate	3%	6%
Kelly intraoperative leak: Large diaphragmatic / dural defect	8%	18%
Postoperative leak	4%	4%
Any postoperative complication	17%	15%

### Associations with preoperative quality of life

The two multivariate logistic regression models classifying the highest and lowest preoperative ASBS quartiles were both significant (LLR p<0.05). High preoperative ASBS scores were significantly associated with three covariates: institution, insulin dependent diabetes (negative association) and lesions at the planum sphenoidale / tuberculum sella anatomic site (Table 2). Patients with insulin-dependent diabetes were five times less likely to report high preoperative ASBS scores than patients without insulin dependent diabetes. Patients with lesions at the planum sphenoidale / tuberculum sella anatomic site were more than five times more likely to report high preoperative ASBS scores than patients with lesions at other anatomic sites.

**Table 2:** Multivariate logistic regression showing associations between covariates and preoperative ASBS quality of life scores (top quartile). *p<0.05. **p<0.01. The MX.X numbers are model codes. M1 stands for model 1, which was designed to detect associations with high preoperative QoL. M1.1 is the first iteration of model 1, M1.2 is the second iteration of model 1, and so on. The MX FULL model contains all covariates.

Covariate	Associations with high preoperative quality of life						Associations with low preoperative quality of life					
	M1.1	M1.2	M1.3	M1.4	M1 FULL		M2.1	M2.2	M2.3	M2.4	M2 FULL	
	Coef.	Coef.	Coef.	Coef.	Coef.	OR (95% CI)	Coef.	Coef.	Coef.	Coef.	Coef.	OR (95% CI)
Intercept	-1.14	-0.69	-0.60	-0.88	-0.65	0.52 (0.11, 2.54)	-1.22	-2.00**	-2.05**	-1.93*	-2.10*	0.12 (0.02, 0.62)
Institution 01	0.06	0.03	-0.06	-0.06	-0.07	0.93 (0.52, 1.68)	-0.28	-0.30	-0.35	-0.44	-0.36	0.7 (0.38, 1.29)
Institution 02	-0.29	-0.47	-0.55	-0.64	-0.78*	0.46 (0.22, 0.94)	-0.23	-0.18	-0.12	-0.05	0.16	1.17 (0.56, 2.46)
Female	-0.15	-0.07	-0.02	-0.06	-0.18	0.83 (0.52, 1.33)	0.63**	0.66**	0.58*	0.58*	0.66**	1.93 (1.18, 3.16)
Age >20 to ≤40	0.13	-0.11	-0.12	-0.23	-0.22	0.8 (0.22, 2.97)	0.16	0.15	0.25	0.12	-0.09	0.92 (0.26, 3.29)
Age >40 to ≤60	0.24	-0.14	-0.10	-0.24	-0.27	0.76 (0.21, 2.8)	-0.24	-0.17	-0.05	-0.13	-0.21	0.81 (0.22, 2.92)
Age >60	0.16	-0.26	-0.11	-0.19	-0.19	0.83 (0.22, 3.07)	-0.12	-0.02	0.02	0.03	-0.10	0.91 (0.25, 3.26)
Presentation history:												
Headaches		-0.22	-0.19	-0.12	-0.09	0.92 (0.53, 1.6)		--	--	--	--	--
Hormonal		-0.40	-0.22	-0.22	-0.16	0.85 (0.39, 1.86)		0.92**	0.76*	0.44	0.40	1.49 (0.71, 3.14)
Visual		-0.10	-0.10	-0.05	-0.22	0.81 (0.42, 1.53)		0.71**	0.78**	0.96**	0.87*	2.38 (1.2, 4.72)
Incidental		0.60	0.54	0.53	0.45	1.57 (0.73, 3.37)		-0.40	-0.26	-0.15	-0.18	0.83 (0.31, 2.25)
Surveillance		0.50	0.39	0.35	0.52	1.69 (0.7, 4.06)		0.43	0.54	0.63	0.33	1.39 (0.54, 3.6)
Rhinorrhea		-0.85	-1.01	-1.08	-0.86	0.42 (0.07, 2.5)		1.27*	1.31*	1.55*	0.91	2.48 (0.6, 10.2)
Other		0.15	0.30	0.29	0.52	1.67 (0.63, 4.45)		0.50	0.37	0.35	0.10	1.11 (0.43, 2.85)
Co-morbidities:												
Atrial fibrillation			0.13	0.10	0.21	1.24 (0.27, 5.66)			-1.77	-1.76	-1.55	0.21 (0.02, 2.34)
Asthma			-0.52	-0.57	-0.61	0.55 (0.1, 2.85)			-0.33	-0.29	-0.19	0.82 (0.19, 3.51)
Cancer (non-CNS)			-0.48	-0.45	-0.45	0.64 (0.12, 3.24)			0.11	0.12	0.04	1.04 (0.3, 3.62)
Depression			-0.87	-0.89	-1.18	0.31 (0.07, 1.45)			-0.22	-0.26	-0.13	0.88 (0.28, 2.8)
Diabetes (non-insulin requiring)			-0.08	-0.09	0.12	1.12 (0.44, 2.88)			0.81	0.72	0.62	1.87 (0.77, 4.51)
Diabetes (insulin-requiring)			-1.50*	-1.51*	-1.81**	0.16 (0.04, 0.64)			1.37**	1.18**	1.13**	3.11 (1.35, 7.14)
Hypertension			-0.25	-0.27	-0.47	0.63 (0.33, 1.18)			-0.39	-0.44	-0.34	0.71 (0.38, 1.31)
High Cholesterol			0.51	0.49	0.74	2.09 (0.92, 4.75)			-0.68	-0.68	-0.78	0.46 (0.18, 1.16)
Osteoarthritis			-0.41	-0.33	-0.32	0.72 (0.13, 3.88)			1.02	0.81	0.77	2.16 (0.52, 8.94)
Ulcer disease			-1.18	-1.15	-1.07	0.34 (0.02, 6.22)			1.03	1.08	0.83	2.29 (0.43, 12.26)
Secreting adenoma				0.56	0.48	1.61 (0.71, 3.67)				0.58	0.94*	2.56 (1.12, 5.85)
Normal preoperative endocrine function				0.52	0.50	1.65 (0.89, 3.06)				-0.31	-0.33	0.72 (0.37, 1.37)
Reoperation					-0.29	0.75 (0.36, 1.57)					0.41	1.5 (0.74, 3.05)
Anatomic site:												
Sella					0.01	1.01 (0.49, 2.08)					-0.28	0.75 (0.34, 1.65)
Suprasellar cistern					-0.16	0.85 (0.47, 1.56)					0.60	1.82 (0.92, 3.61)
Clivus					0.02	1.02 (0.41, 2.55)					0.71	2.03 (0.79, 5.21)
Planum sphenoidale / tuberculum sella					1.65**	5.22 (2.19, 12.45)					-0.59	0.55 (0.2, 1.51)
Anterior fossa / olfactory groove					-0.58	0.56 (0.16, 1.96)					0.69	1.99 (0.64, 6.17)
Sphenoid sinus					-0.04	0.96 (0.39, 2.35)					-0.08	0.92 (0.36, 2.38)
Ethmoid sinus					-1.20	0.3 (0.07, 1.24)					0.72	2.05 (0.58, 7.28)
Cavernous sinus					0.21	1.24 (0.49, 3.14)					-1.33*	0.26 (0.07, 0.94)

Low preoperative ASBS scores were significantly associated with five covariates. There were positive associations with female gender, a vision-related presentation history, insulin-dependent diabetes and secreting adenoma. A negative association was found for lesions at the cavernous sinus anatomic site (Table 2). Women were almost two times more likely to experience QoL scores in the lowest quartile than men. Patients with a vision-related presentation history were more than twice as likely to report preoperative QoL scores in the lowest quartile than patients without a vision related presentation history. Patients with insulin-dependent diabetes were more than three times more likely to report preoperative QoL scores in the lowest quartile than patients without insulin-dependent diabetes. Patients with lesions in the cavernous sinus were four times less likely to report low preoperative ASBS scores than patients with lesions at other anatomic sites. The strength of the association between insulin dependent diabetes and QoL was highlighted by the significant positive association with low preoperative ASBS and the significant negative association with high preoperative ASBS scores.

### Associations with top quartile change in quality of life at 12-month follow-up

The multivariate logistic regression model predicting top quartile change in postoperative ASBS scores at 12 months was significant (Table 3). Five covariates were significantly associated with top quartile change in postoperative ASBS at 12-month follow-up. Patients who presented with high preoperative cholesterol and acromegaly were less likely to experience a substantial positive change in QoL at 12 months. Patients with a lesion at the sphenoid sinus anatomic site were almost five times more likely to experience a substantial positive change in QoL at 12 months than patients with lesions at other anatomic sites. Patients with deficient preoperative endocrine function were four times more likely to experience a substantial positive change in QoL at 12 months than other patients. Patients who experienced a Grade 3 intraoperative CSF leak (large diaphragmatic/dural defect) were more than six times less likely to experience a substantial positive change in QoL at 12 months compared to other patients. The logistic regression model classifying the lowest QoL quartile at 12 months was not significant.

**Table 3:** Multivariate logistic regression showing associations between covariates and high positive changes in postoperative ASBS quality of life scores at 12 months (highest quartile). *p<0.05. **p<0.01. SIADH, syndrome of inappropriate antidiuretic hormone secretion. The MX.X numbers are model codes. M3.1 is the first iteration of model 3, M3.2 is the second iteration of model 3, and so on. The M3 FULL model contains all covariates.

Covariate	Predicting top quartile change in postoperative ASBS score at 12 months					
	M3.1	M3.2	M3.3	M3.4	M3 FULL
	Coef.	Coef.	Coef.	Coef.	Coef.	OR (95% CI)
Intercept	-1.29	-1.09	-1.14	-1.08	-1.42	0.24 (0.03, 1.92)
Institution 01	1.00*	0.81*	0.75*	0.71	0.61	1.85 (0.76, 4.5)
Age >20 to ≤40	-1.14	-1.11	-1.09	-0.99	-1.09	0.33 (0.05, 2.34)
Age >40 to ≤60	0.00	0.08	0.08	0.51	0.42	1.52 (0.24, 9.78)
Age >60	-0.29	-0.10	-0.05	0.04	-0.27	0.77 (0.12, 4.88)
Presentation history:						
Headaches		-0.01	-0.02	-0.10	-0.14	0.87 (0.35, 2.18)
Incidental		-0.28	-0.25	-0.89	-0.82	0.44 (0.13, 1.51)
Surveillance		-0.44	-0.59	-1.02	-0.37	0.69 (0.11, 4.25)
Co-morbidities:						
Diabetes (non-insulin requiring)		-0.60	-0.79	-0.84	-1.21	0.3 (0.04, 2.14)
Diabetes (insulin-requiring)		-0.02	-0.37	-0.21	-0.44	0.64 (0.07, 5.66)
High Cholesterol		-1.09	-1.12	-1.81*	-2.03*	0.13 (0.02, 0.85)
Anatomic site:						
Planum sphenoidale / tuberculum sella			-0.49	0.58	1.48	4.37 (0.46, 41.87)
Anterior fossa / olfactory groove			-0.02	0.46	1.23	3.42 (0.34, 34.23)
Sphenoid sinus			1.10	0.90	1.56*	4.76 (1.26, 17.96)
Histopathology:						
Acromegaly				-2.36	-2.76*	0.06 (0.01, 0.79)
Gonadotroph secretory pituitary adenoma				-1.17	-1.40	0.25 (0.05, 1.33)
Meningioma				-3.19	-2.31	0.1 (0, 6.02)
Non-functioning pituitary adenoma				0.70	0.75	2.11 (0.73, 6.11)
Pituitary cyst-Rathke’s cleft cyst				-2.19	-2.57	0.08 (0, 3.23)
Preoperative endocrine function deficient					1.40*	4.06 (1.23, 13.35)
Preoperative endocrine function hypersecreting					0.79	2.2 (0.69, 6.95)
Postoperative development of diabetes insipidus					-0.50	0.61 (0.15, 2.44)
Postoperative development of SIADH					-0.99	0.37 (0.04, 3.24)
Resection: subtotal / debulk					0.71	2.03 (0.69, 5.99)
Kelly intraoperative leak: Moderate					0.30	1.35 (0.23, 8.12)
Kelly intraoperative leak: Large diaphragmatic / dural defect					-1.93*	0.15 (0.03, 0.73)
Postoperative leak					-0.71	0.49 (0.03, 8.56)
Any postoperative complication					1.07	2.93 (0.77, 11.08)

### Training supervised learning models to predict improvement in quality of life at 12-month follow-up

**Fig 2:**
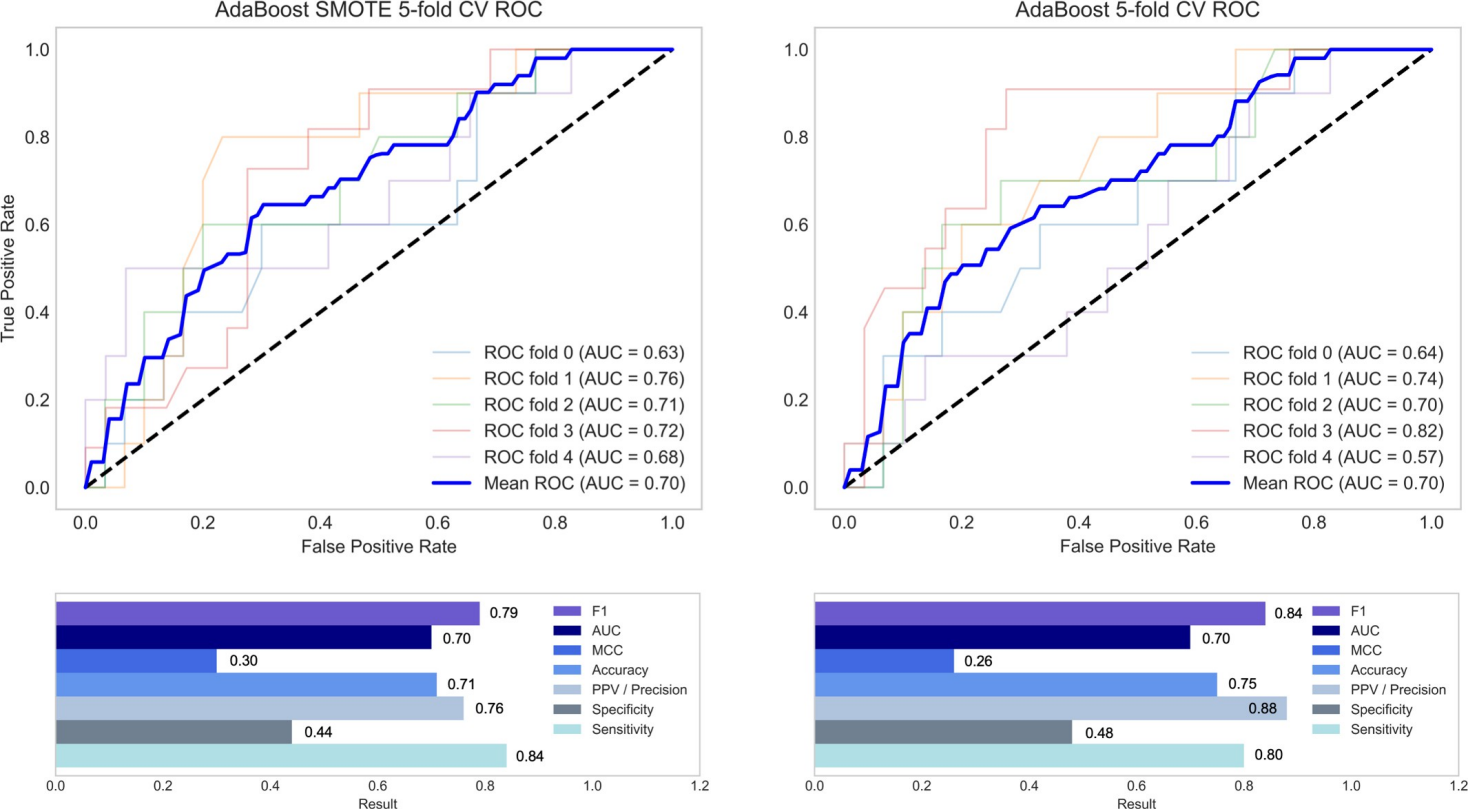
Mean 5-fold cross-validation ROC curves and performance metrics for the top performing classifiers used to predict top quartile change in postoperative ASBS score at 12 months.

**Fig 3:**
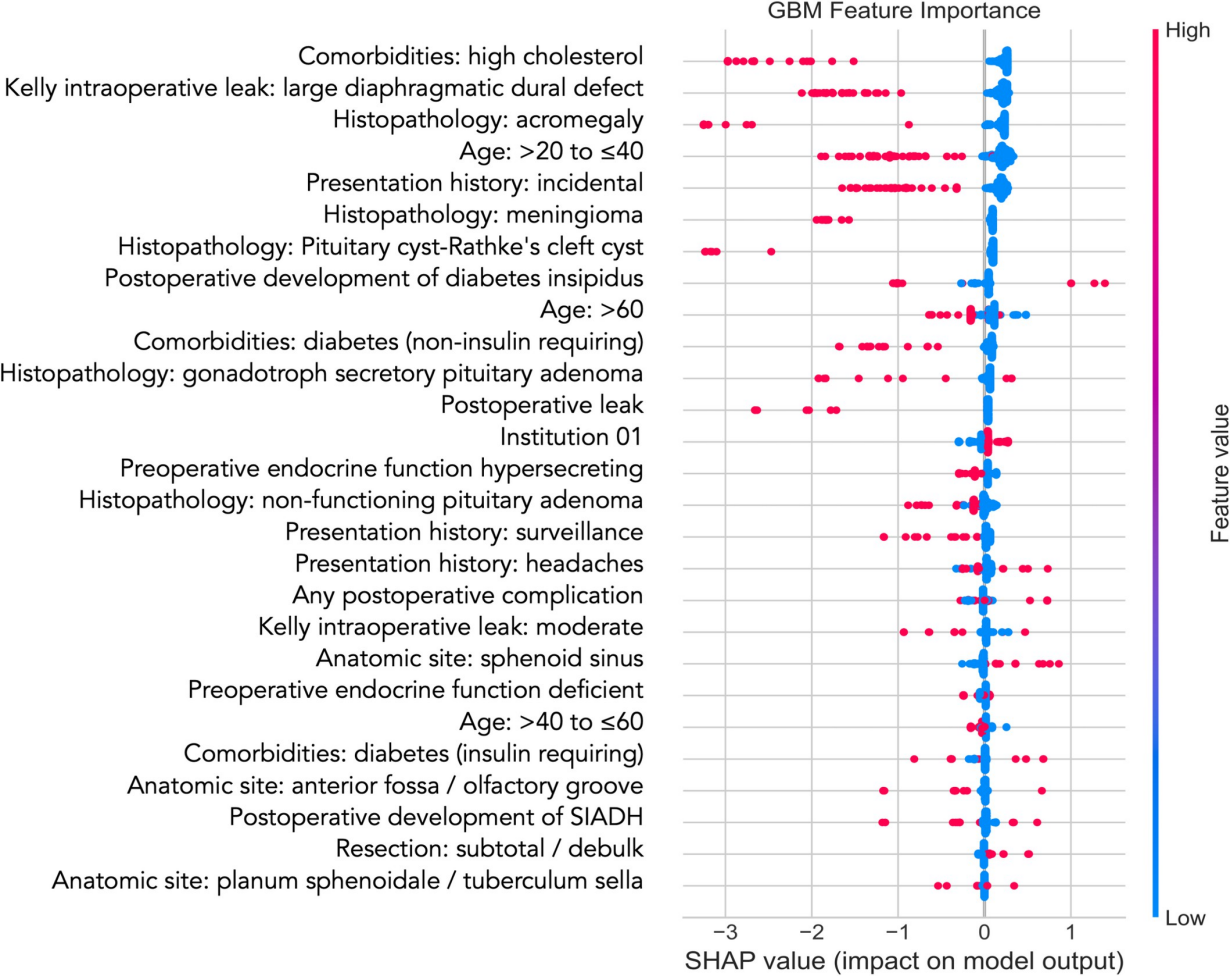
Gradient boosting machine (GBM) feature importance. SHAP, Shapley additive explanations. SIADH, syndrome of inappropriate antidiuretic hormone secretion.

Supervised learning models were trained using two groups of covariates: (1) statistically selected covariates from the preceding multivariate logistic regression analysis, and (2) the top 27 most relevant covariates selected using RFE. Mean five-fold cross-validation performance metrics for each classifier are displayed in Table 4, sorted by MCC. AdaBoost, logistic regression and neural network classifiers demonstrated the strongest performance. Across all algorithms, the application of SMOTE to the training dataset resulted in significantly lower precision, accuracy and AUC on the holdout test set (Table 5). Features selected statistically using multivariate logistic regression resulted in significantly higher AUC and MCC across all algorithms when compared with RFE (p<0.05). Fig 2 presents ROC curves and performance metrics for top performing classifiers. The SHAP summary plot (Fig 3) presents relationships between covariates and the outcome variable in the highest performing GBM model. SHAP relationships were consistent with the statistical associations demonstrated by multivariate logistic regression. A blended ensemble approach did not yield classification performance improvements. Models designed to predict the lowest quartile change in ASBS at 12 months did not yield acceptable performance results.

**Table 4:** Mean 5-fold cross-validation algorithm performance results, sorted by MCC. SMOTE was applied to the training dataset only. GBM, gradient boosting machine. LR, logistic regression. NB, naïve Bayes. RFE, recursive feature elimination. SMOTE, synthetic minority oversampling technique. SVM, support vector machine.

Algorithm	Features	Data augmentation	MCC	AUC	Sensitivity / Recall	Specificity	PPV / Precision	Accuracy	F1
AdaBoost	LR statistical	SMOTE	0.30	0.70	0.84	0.44	0.76	0.71	0.79
AdaBoost	LR statistical	No SMOTE	0.26	0.70	0.80	0.48	0.88	0.75	0.84
Logistic regression	LR statistical	SMOTE	0.25	0.68	0.83	0.40	0.70	0.67	0.76
Neural network	RFE	No SMOTE	0.24	0.62	0.78	0.54	0.93	0.75	0.85
Neural network	LR statistical	No SMOTE	0.23	0.65	0.81	0.43	0.81	0.71	0.81
Random forest	RFE	No SMOTE	0.22	0.66	0.77	0.57	0.95	0.75	0.85
Neural network	LR statistical	SMOTE	0.21	0.64	0.81	0.39	0.73	0.67	0.76
Random forest	LR statistical	SMOTE	0.20	0.65	0.80	0.38	0.74	0.67	0.77
GBM	RFE	No SMOTE	0.18	0.65	0.77	0.47	0.90	0.73	0.83
SVM	LR statistical	SMOTE	0.18	0.65	0.79	0.39	0.80	0.69	0.80
GBM	LR statistical	SMOTE	0.18	0.63	0.80	0.36	0.70	0.65	0.75
K-nearest neighbor	LR statistical	No SMOTE	0.17	0.62	0.77	0.55	0.93	0.74	0.84
Logistic regression	LR statistical	No SMOTE	0.16	0.71	0.77	0.00	0.97	0.76	0.86
K-nearest neighbor	RFE	SMOTE	0.16	0.57	0.78	0.42	0.86	0.71	0.82
K-nearest neighbor	LR statistical	SMOTE	0.15	0.63	0.81	0.32	0.57	0.58	0.67
Gaussian NB	LR statistical	SMOTE	0.14	0.65	0.81	0.31	0.49	0.54	0.61
Random forest	LR statistical	No SMOTE	0.14	0.63	0.78	0.34	0.89	0.73	0.83
AdaBoost	RFE	No SMOTE	0.13	0.64	0.77	0.40	0.89	0.72	0.82
Gaussian NB	LR statistical	No SMOTE	0.13	0.64	0.88	0.28	0.19	0.38	0.31
Logistic regression	RFE	No SMOTE	0.11	0.65	0.76	0.00	0.97	0.75	0.85
Logistic regression	RFE	SMOTE	0.10	0.59	0.79	0.29	0.49	0.52	0.60
AdaBoost	RFE	SMOTE	0.09	0.61	0.79	0.29	0.48	0.52	0.59
K-nearest neighbor	RFE	No SMOTE	0.09	0.57	0.76	0.40	0.96	0.74	0.85
Neural network	RFE	SMOTE	0.08	0.60	0.77	0.31	0.51	0.53	0.60
GBM	LR statistical	No SMOTE	0.06	0.66	0.76	0.31	0.85	0.69	0.80
Gaussian NB	RFE	No SMOTE	0.06	0.65	0.00	0.25	0.45	0.50	0.00
SVM	RFE	No SMOTE	0.05	0.65	0.75	0.00	0.92	0.71	0.83
Random forest	RFE	SMOTE	0.05	0.59	0.77	0.28	0.51	0.52	0.60
Gaussian NB	RFE	SMOTE	0.05	0.58	0.82	0.22	0.36	0.45	0.42
GBM	RFE	SMOTE	0.02	0.57	0.74	0.28	0.49	0.50	0.58
SVM	LR statistical	No SMOTE	-0.01	0.68	0.74	0.00	0.97	0.73	0.84
SVM	RFE	SMOTE	-0.02	0.50	0.74	0.25	0.51	0.50	0.59

**Table 5:** Differences in mean performance results across all applied algorithms. AUC, area under the receiver operating characteristic curve. MCC, Matthews correlation coefficient. ns, not significant. RFE, recursive feature elimination. SD, standard deviation. SMOTE, synthetic minority oversampling technique.

	Unaugmented data (mean, SD)	SMOTE on training set (mean, SD)	Significance	Logistic regression features (mean, SD)	RFE features (mean, SD)	Significance
Sensitivity	0.73 (0.20)	0.79 (0.03)	ns	0.80 (0.03)	0.72 (0.19)	ns
Specificity	0.31 (0.21)	0.33 (0.07)	ns	0.34 (0.15)	0.31 (0.16)	ns
Precision	0.84 (0.21)	0.61 (0.15)	p<0.01	0.75 (0.20)	0.70 (0.23)	ns
Accuracy	0.70 (0.11)	0.59 (0.09)	p<0.01	0.67 (0.10)	0.62 (0.12)	ns
MCC	0.14 (0.08)	0.13 (0.09)	ns	0.17 (0.08)	0.10 (0.07)	p<0.05
F1	0.75 (0.24)	0.67 (0.11)	ns	0.75 (0.14)	0.67 (0.23)	ns
AUC	0.65 (0.03)	0.62 (0.05)	p<0.05	0.66 (0.03)	0.61 (0.04)	p<0.01

## Discussion

This multi-institutional study was designed to (1) determine associations between perioperative clinical factors and (a) preoperative QoL and (b) postoperative improvement in QoL in patients undergoing anterior endoscopic skull base surgery and (2) train supervised learning classifiers to predict postoperative change in QoL. Change in QoL 12 months after endoscopic skull base surgery in this sample was, on average, significant and positive. Mean change in ASBS score at 12-month follow up (7.5) was much higher than the established minimally important clinical difference (0.4) [60], suggesting that endoscopic skull base surgery on average yielded substantial and clinically important QoL improvements for patients responding at 12-month follow-up. Multiple significant associations were detected between clinical covariates and QoL scores, controlling for demographics, comorbidities, lesion anatomic site, histopathology and various other perioperative factors. These associations may facilitate treatment planning, understanding of clinical mechanisms and clinical decision making. Machine learning models demonstrated moderate predictive performance. AdaBoost, neural network and logistic regression classifiers demonstrated the highest predictive performance as measured by the MCC, F1 and AUC metrics. Models may be further refined and improved, externally validated and considered for deployment in practice as clinical decision support tools. Accurately predicting postoperative improvement in QoL may facilitate treatment decision making and recovery planning for clinicians and patients. Appropriately implemented supervised learning models have the potential to improve the informed consent process, healthcare efficiency, care quality and patient safety [14, 15, 61]. Routine assessment of QoL for patients with pituitary tumours, both before and after treatment, has been recommended [3]. This recommendation may be extended to incorporate the application of high performing predictive models to forecast and optimise future QoL. The utility of appropriately developed supervised learning-based decision support systems for neurosurgeons and their patients is becoming clearer [14, 16, 25, 62].

Statistical associations detected using multivariate logistic regression were well supported by existing literature. For example, lower cholesterol has previously been associated with higher QoL [63] and endocrinopathy [64, 65] and female gender [66] have previously been associated with lower QoL amongst neurosurgery patients. Diabetes was strongly and negatively associated with QoL in this patient sample, reinforcing a well-established relationship [67]. QoL consists of cognitive and emotional components (e.g., satisfaction and happiness) [68] and multiple factors modify QoL in patients with diabetes, including medication adherence, disease duration, depression, insulin use, and the presence of comorbidities [69–72]. People with diabetes often feel burdened by the management demands of their disease and lower mood has been associated with higher HbA1c levels [73]. The complications of diabetes have a negative emotional and physical impact on patients and are associated with wellbeing decrements [74, 75].

It appears that QoL was influenced by lesion anatomic site. Patients with a lesion at the cavernous sinus site were less likely to report low preoperative QoL, while patients with a lesion at the planum sphenoidale / tuberculum sella site were much more likely to report high preoperative QoL than patients with lesions at other sites. Together, these results suggest that, overall, lesions at these sites tended to be associated with higher preoperative QoL. Patients with sphenoid sinus lesions were more likely to experience a positive change in postoperative QoL at 12 months. There may be numerous factors (e.g., location accessibility, ease of lesion resection, corresponding endocrinopathy, or impingement on adjacent anatomical structures, etc.) associated with lesions at some sites that may influence preoperative QoL and make them more amenable to surgical treatment, resulting in more substantial QoL improvements. Lesions at the planum sphenoidale tend to be meningiomas, which are typically benign and usually cause visual disturbance rather than endocrine dysfunction [76]. This may explain an increased likelihood of higher preoperative QoL in patients with lesions at this anatomic site. Similarly, tumours in the cavernous sinus tend to be benign and responsive to simple symptomatic treatment [77], which may explain the negative relationship between lesions at this anatomic site and low preoperative QoL.

Overall, the highest performing machine learning classifiers yielded a moderate level of classification performance. These classifiers, nevertheless, demonstrated performance that was better than chance and as such may offer an additional useful input into the clinical decision-making process. We have, in this work, presented a benchmark for the field using some standard methods and a modest dataset. Interestingly, classifier performance appeared to be significantly affected by the dimensionality reduction and data augmentation methods applied. Dimensionality reduction using multivariate logistic regression appeared to yield superior classifier performance when compared with RFE. Furthermore, data augmentation applied as recommended [47] did not yield superior classifier performance results in this study, which casts doubt on the utility of SMOTE when working with small clinical datasets. These results may be useful to machine learning practitioners and beneficially inform future clinically applied machine learning work.

A salient issue in the field of clinically applied machine learning and machine ethics, which was intentionally addressed, is that of model interpretability or explainability [78]. Neural networks are opaque, trading higher performance for poor interpretability. Coupling neural networks and other inscrutable classifiers with multivariate logistic regression, which presents statistical association information for each covariate in the model, helps to facilitate interpretability, clinician understanding and trust [16]. Deploying tree-based models with SHAP analysis is another technique that further facilitates interpretability [31]. Both techniques were applied in this project to promote clinical insight, understanding and model utility.

## Limitations and future research

Future research may consider the development of refined high-performance models that contain less covariates to facilitate more efficient system implementation, usability and generalisability. Analyses based on larger sample sizes from multiple institutions would facilitate a more detailed investigation of QoL associations at additional postoperative time points. Larger datasets would also allow for the use of one or more holdout datasets to more rigorously evaluate and validate classifier performance. The number of ASBS respondents at 12 months was lower than the number of preoperative respondents. Verification of results through replication and external validation is required as selection bias may have influenced results. Specialist clinical datasets are difficult and expensive to acquire and maximal use of data for beneficial research is an ethical issue. The research team is exploring the use of alternative classifier implementations that allow for missing data.

## Conclusion

Significant associations were detected between perioperative clinical factors, preoperative QoL scores and improvement in postoperative QoL scores at 12 months amongst patients undergoing anterior endoscopic skull base surgery. This study demonstrated that machine learning may be applied to predict changes in QoL at 12-month follow-up using perioperative data, facilitating optimisation of patient care and outcomes.

## Supporting information

S1 Checklist TRIPOD checklist: Prediction model development and validation. (PDF)
